# Structural Characterization of Graphene Oxide: Surface Functional Groups and Fractionated Oxidative Debris

**DOI:** 10.3390/nano9081180

**Published:** 2019-08-18

**Authors:** Elvin Aliyev, Volkan Filiz, Muntazim M. Khan, Young Joo Lee, Clarissa Abetz, Volker Abetz

**Affiliations:** 1 Helmholtz-Zentrum Geesthacht, Institute of Polymer Research, Max-Planck-Str. 1, 21502 Geesthacht, Germany; 2 Institute of Inorganic and Applied Chemistry, Department of Chemistry, University of Hamburg, Martin-Luther-King-Platz-6, 20146 Hamburg, Germany; 3 Institute of Physical Chemistry, Department of Chemistry, University of Hamburg, Martin-Luther-King-Platz-6, 20146 Hamburg, Germany

**Keywords:** graphene oxide, base-washed graphene oxide, surface functional group analysis, oxidative debris, standardized Boehm titration method

## Abstract

The purpose of this work is the structural analysis of graphene oxide (GO) and by means of a new structural model to answer the questions arising from the Lerf–Klinowski and the Lee structural models. Surface functional groups of GO layers and the oxidative debris (OD) stacked on them were investigated after OD was extracted. Analysis was performed successfully using Fourier transform infrared spectroscopy (FTIR), ultraviolet-visible spectroscopy (UV-Vis), X-ray photoemission spectroscopy (XPS), energy-dispersive X-ray spectroscopy (EDX), Raman spectroscopy, solid-state nuclear magnetic resonance spectroscopy (SSNMR), standardized Boehm potentiometric titration analysis, elemental analysis, X-ray diffraction (XRD), thermogravimetric analysis (TGA), scanning electron microscopy (SEM), and transmission electron microscopy (TEM). The analysis showed that graphene oxide layers, as well as oxidative debris contain different functional groups such as phenolic –OH, ketone, lactone, carboxyl, quinone and epoxy. Based on these results, a new structural model for GO layers is proposed, which covers all spectroscopic data and explains the presence of the other oxygen functionalities besides carboxyl, phenolic –OH and epoxy groups.

## 1. Introduction

Having unique properties, graphene layers are a quite interesting nanomaterial for industrial applications. However, the generation of graphene layers from graphite is quite expensive, but can be achieved at lower cost via controlled oxidation of graphite [1]. Graphene oxide (GO) layers are the individual sheets of conventional graphite oxide (CGO) synthesized from graphene layers of graphite. 

Oxidation of graphite leads to the formation of GO whose structure and the mechanism of formation are still unclear and need further investigation. The structural examination of GO is a necessary step for further functionalization, for the reduction and for determining the structure of its origin-graphene. This helps to reduce the manufacturing expenses of graphene production and to synthesize new types of graphene nanoparticles for future applications such as lithography, biosensoring, drug delivery, etc. [2,3]. Recent progress in membrane science demands new types of nanomaterials that could be used as a nanofiller in thin-film composite membranes in the field of gas separation and water purification. Graphene oxide is an excellent material to solve this problem [4,5,6]; however, structural investigation can help to understand the percolation threshold of graphene oxide layers in mixed matrix and to predict the molecular transport phenomenon in the membranes.

Up to now, several structural models [7,8,9,10,11] have been proposed for graphene oxide nanosheets with different oxygen containing functional groups. The starting materials and the oxidation conditions determine the composition of GO. The main oxygen containing functional groups, which are distributed on GO nanosheets are epoxides (C–O–C), phenolic hydroxyl (–OH), carboxylic (–COOH), and other carbonyl groups (C=O) resulting in polar surface properties. Due to these functionalities, GO nanosheets are hydrophilic and exfoliate easily in aqueous media [12]. It is commonly accepted that on GO nanosheets carboxylic (–COOH) groups are mainly located at the edges, while phenolic hydroxyl (–OH) and epoxy (C–O–C) groups are located in the basal plane [13]. During oxidation of the graphene layers of graphite, defects are generated and oxidation occurs at these defect sites [14]. Theoretically, the main possible oxygen containing functionalities at defect sites are mainly ketone and quinone type groups; however, depending on the size of the defects, other functional groups can also be formed.

The oxidation process also activates a side product formation during GO synthesis. Research on carbon nanotubes has revealed that during oxidation of the nanocarbons oxidative debris (OD) is generated and adsorbs on the nanotubes [15]. OD influences the stability of GO nanosheets significantly in the suspension [16] and causes a yellow color. However, there is little information available about OD acting as a natural surfactant for the stability of the GO nanosheets in dispersions and the effect on agglomeration of graphene oxide layers [17]. Therefore, the structural investigation of OD helps to understand the structure of large graphene oxide layers.

To the best of our knowledge, so far the proposed structural models for GO leave some open questions. The Lerf–Klinowski model—the most cited model for GO—does not give answers to the following problems: (a) in this model, the carboxyl groups are shown at the edges even though the NMR data do not support the existence of this group, and (b) the hydroxyl groups are located close to each other, leading to the suspicion that there is an electrical instability in the structure [10,11]. The Lee model proposed in 2010 does not include the carboxyl and lactone groups in the structure, although the solid-state ^13^C NMR data shows the existence of both groups [11].

In this paper, we try to uncover the formation of GO and OD, and to analyze the structure of both and to propose a new structural model for GO layers. The model explains the origin of lactone peaks, the presence of defects and quinone groups, which have never been discussed in the literature before. The experiments showed that during synthesis OD is generated, which increases the difficulties in terms of functional group determination of GO. For successful analysis, OD is extracted from GO layers and precipitated by the Hiradate method [18]. This method consists of the adsorption of OD on a hydrophobic resin (DAX-8) followed by complete elution using acidic, neutral and basic solutions sequentially.

The proposed structure will help to modify the Lerf–Klinowski model and open new directions in the field of graphene-like nanomaterials synthesis.

## 2. Materials and Methods

### 2.1. Materials

Graphite (natural, crystallic, briquetting grade, −100 mesh, 99.9995%, Ultra “F” purity), sulfuric acid (H_2_SO_4_, ACS, 95.0–98.0%), potassium permanganate (KMnO_4_, ACS, 99.0%) and potassium hydrogen phthalate (C_8_H_5_KO_4_, primary standard, ACS, 99.95–100.05%) were purchased from Alfa Aesar (Heysham, UK). Sodium nitrate (NaNO_3_, ACS, 99.5%), sodium carbonate (Na_2_CO_3_, anhydrous grade for analysis, 99.9%), sodium hydroxide solution (NaOH, Titrisol, 1 M) and hydrogen chloride (HCl, reagent grade for analysis, 37%) were purchased from Merck (Kenilworth, NJ, USA. Milli-Q ultrapure water (>18 MΩ·cm at 25 °C) and aluminum chloride (AlCl_3_, anhydrous powder sublimed for synthesis, 98.0%) were purchased from Merck Millipore (Darmstadt, Germany). Hydrogen peroxide (H_2_O_2_, Ph. Eur. Stabilized, 30%) was purchased from Carl Roth (Karlsruhe, Germany). Hydrazine monohydrate (N_2_H_4_·H_2_O, reagent grade, 98%), sodium bicarbonate (NaHCO_3_ crystalline, reagent grade, 99.5%), parafilm^®^, DAX-8 resin and 50WX2-100 Dowex ion exchanger were purchased from Sigma-Aldrich (St. Louis, Missouri, USA). 

### 2.2. Synthesis of Conventional Graphite Oxide (CGO)

Conventional graphite oxide (CGO) was synthesized from graphite (4.0 g) by Hummers method [19] in concentrated sulfuric acid (92 mL) using sodium nitrate (2.0 g) and potassium permanganate (12.0 g). The obtained brownish-grey paste was diluted with Milli-Q ultrapure water (>18 MΩ·cm at 25 °C, 184 mL) and treated with hydrogen peroxide (80 mL). Synthesized CGO was vacuum-filtered through qualitative Grade 1 filter paper (pore size 11 μm, Whatman, Maidstone, UK), washed, centrifuged at the speed of 11,000 rpm on a Sigma 6-16 K machine (Sigma Laborzentrifugen GmbH, Osterode am Harz, Germany) and freeze-dried on a Gamma 1–16 LSC plus machine (Martin Christ Gefriertrocknungsanlagen GmbH, Osterode am Harz, Germany).

### 2.3. Preparation of Base-Washed Graphene Oxide (bwGO)

CGO was dispersed in 1.0 M NaOH, shaken for 3 hours, refluxed for an hour at 80 °C, vacuum-filtered in order to remove ~30 wt.% oxidative debris (OD) from the CGO dispersion and after the filter cake was washed until a neutral pH, it was freeze-dried on a Gamma 1-16 LSC plus machine. Then, the Na-form of the base-washed graphene oxide (bwGO) was re-dispersed in 1.0 M HCl solution and refluxed at 80 °C for an hour to regenerate the acidic groups [17,20].

### 2.4. Synthesis of the Reduced Graphene Oxide Forms (Reduced GO (rGO), and Base-Washed and Reduced GO (rbwGO))

2.0 grams of CGO and 2.0 grams of bwGO were separately dispersed in ultrapure water (1.0 L) in two different flasks, sonicated and reacted with hydrazine monohydrate (10 mL) at 100 °C for 24 hours [21].

### 2.5. Surface Functional Groups Determination

The general titration procedure was carried out by a “standardized” Boehm titration method [22,23]. A known mass of approximately 0.01 g of GO samples was added to 25.0 mL of one of three 0.05 M reaction bases: sodium bicarbonate (NaHCO_3_), sodium carbonate (Na2CO3), and sodium hydroxide solution (NaOH,). The vials were nitrogen-purged for 2 hours, sealed and placed in a shaking incubator (Incutec K30-300, EquipNet, Canton, MA, USA, at ambient temperature) at 90 l/min speed for 24 hours. Samples were then filtered through qualitative Grade 1 filter paper (Whatman, pore size 11 μm) and 10 ± 0.02 mL aliquots were taken by pipette from the solutions. The aliquots of the reaction bases NaHCO_3_ and NaOH were acidified by 20 mL 0.05 M HCl (reagent grade for analysis, 37%, Merck). However, the aliquots of Na_2_CO_3_ were acidified by the addition of 30 mL 0.05 M HCl solution. During titration, all aliquots are covered with a seal of parafilm^®^, which maintained around the electrode and burette, and bubbled continuously with nitrogen flow to ensure sufficient removal of CO_2_. 0.05 M NaOH solution was used as a titration base for these experiments and the correctness of NaOH was controlled by potassium hydrogen phthalate (0.2 g) solution in 20 mL Milli-Q ultrapure water (>18 MΩ·cm at 25 °C). Blank samples were also titrated for the accuracy of the measurements.

### 2.6. Exfoliation Experiments of Graphene Oxide and Base-Washed Graphene Oxide Samples.

Exfoliation experiments were conducted both for OD-containing graphene oxide and OD-stripped graphene oxide samples, base-washed GO (bwGO), in order to detect the effect of oxidative debris on agglomeration of graphene layers. 10 aqueous dispersions of both graphene samples were prepared ranging from 0.002 wt.% to 0.1 wt.% and their UV absorbance was analyzed on an UVmini-1240 spectrophotometer at 600 nm after sonication in a bath. According to the result the correlation curves were plotted (Figure S8). Using correlation curves, we were able to determine the concentration of the single layer containing graphene oxide dispersion. 

### 2.7. Isolation, Fractionation and Purification of OD Using a Precipitation Procedure

OD was isolated, fractionated and purified by methods shown elsewhere [18,20]. The filtrate, which contains OD was acidified until pH 1.0 from pH 14.0 with 4.0 M HCl aqueous solution and poured into a glass column (25 mm internal diameter, 650 mm length) prefilled with ca. 300 mL DAX-8 resin. The eluted non-adsorbed fraction was collected, adjusted to pH 5.0 and treated with 0.1 M AlCl_3_ solution to precipitate the colored solution resulting in a clear supernatant. Then, the white dispersion was passed through a 50WX2-100 Dowex ion exchanger column to regenerate the acidic groups. The slimy yellowish sediment was separated from the solution via a separator funnel and freeze-dried (OD-1). The column was eluted successively with 1 L of 0.1 M HCl, 1 L of ultrapure water and 500 mL of 0.1 M NaOH sequentially. All fractions were treated by passing through a 50WX2-100 Dowex ion exchanger corresponding to OD-1 and labelled as OD-2, OD-3 and OD-4, respectively.

### 2.8. Methods

The XPS analysis of the graphene oxide samples was carried out on a Kratos AXIS Ultra DLD spectrophotometer (Kratos, Manchester, UK) using an Al-Kα X-ray source operated at 225 W under ultra-high vacuum (UHV, <2.5·10^−9^ Torr.). Before the experiments, the pre-load chamber was degassed and the graphene oxide samples positioned in the UHV analytics chamber. The analysis were conducted in the area of 700 µm × 300 µm with an acceleration depth of app. 5 nm. All the spectra were calibrated to the C1s signal maintained at 284.5 eV. For the evaluation and validation of the data, CASA-XPS version 2.3.18 was used. Before calculation, the background subtraction (linear or Shirley) was applied for the deconvolution of the different regions of the files. Fourier transform infrared (FTIR) spectra were recorded in attenuated total reflectance (ATR) mode on a Bruker ALPHA FT-IR spectrometer (Bruker, Ettlingen, Germany). The transmittance measurements were collected at ambient temperature in a spectral range of 400–4000 cm^−1^ at a resolution of 4 cm^−1^ and average of 64 scans. UV-Vis spectra were collected on an UVmini-1240 spectrophotometer (Shimadzu, Kyoto, Japan) in the absorbance mode with a 10 mm cuvette in the wavelength range of 190–1100 nm applying 0.02 wt.% solutions. Solid-state NMR (SSNMR) experiments were performed on a Bruker Avance II 400 spectrometer (Bruker, Rheinstetten, Germany) equipped with a 4 mm double resonance probe. Direct excitation ^13^C magic angle spinning (MAS) NMR spectra were obtained with a 45° pulse length of 2.05 μs and recycle delay of 30 s at an operating frequency of 100.66 MHz. ^13^C{^1^H} cross polarization (CP) MAS spectra were acquired using ramped polarization transfer with a ^1^H 90° pulse length of 4.0 µs, contact time of 1 ms, and a repetition delay of 2 s. Two-pulse phase-modulated (TPPM) decoupling was used during the acquisition for both experiments. All the experiments were conducted with a spinning frequency of 13 kHz at room temperatures. Elemental analysis (EA) was carried out with a EuroEA Elementar CHNSO Analyser (EuroVector, Pavia, Italy). The amounts of carbon, hydrogen, nitrogen, and oxygen were quantitatively determined by the dry combustion method. The standardized Boehm titration method was carried out on a fully automatized Titrino Plus 848 (Metrohm, Filderstadt, Germany) equipped with a 20 mL burette using a 0.05 M NaOH solution. Before experiments, the NaOH solution was standardized with potassium hydrogen phthalate. Raman spectra were obtained using a Senterra (Bruker, Ettlingen, Germany) Raman spectrometer equipped with a 532 nm excitation laser and 10 fold objective lens. The results were estimated by extracting each single spectrum and the areas corresponding to the D mode (disorder induced mode, centered around 1300 cm^−1^) and the G mode (graphite mode, around 1550 cm^−1^) have been evaluated by two Gaussian fits. A D8 discover X-ray diffractometer (Bruker, Ettlingen, Germany) with Cu Kα radiation (λ = 1.54184 Å, 50 kV, 1000 mA) was applied for the XRD experiments of the graphene oxide samples at a scanning rate of 1° min^−1^. Air-tight sample holders (Bruker, Ettlingen, Germany) were used to prevent any contaminations of the samples. Thermal gravimetric analysis (TGA) was used to investigate the mass loss of functionalized GO samples as a function of temperature. The analysis was carried out on a Netzsch TG209 F1 Iris instrument (Netzsch, Selb, Germany) under argon flow (50 mL min^−1^) from 25 °C to 800 °C at 10 K min^−1^. Scanning electron microscopy experiments were carried out on a Merlin (ZEISS, Oberkochen, Germany). Before investigating the surface and cross section morphology of the samples, they were coated with approx. 2 nm Pt using a sputter coating device MED 020 (Leica Microsystems, Wetzlar, Germany). Secondary electron (SE) images and energy-dispersive X-ray (EDX) spectra were taken at accelerating voltages of 2–3 kV and at 10 kV, respectively. Transmission electron microscopy was carried out on a Tecnai F20 G2 (Thermo Fisher Scientific, Eindhoven, The Netherlands) at 120 kV in the bright field mode. Graphene oxide was diluted in chloroform (0.001wt.%). A droplet of 3 µL was put on a Lacey grid and dried.

## 3. Results and Discussion

The formation of graphene oxide (GO) depends on several factors such as the concentration of acids, the strength of oxidizer and the decomposition of the intermediate compounds. By following Hummers method, we used highly concentrated sulfuric acid (18 M) and sodium nitrate. The first intercalation occurs immediately by sulfuric acid after adding graphite, graphite bisulfate generates [24] and this state of graphite is called stage-1 GIC (graphite-intercalated compound). While HSO_4_^-^ ions attack the edge of the graphene layers, Na^+^ ions enter between graphene layers removing Van der Waals and π-π stacking interactions, and the *d*-spacing increases between the layers. The next intercalation, after expanding the graphene layers, occurs on basal planes. Simultaneously, nitric acid is generated in the sulfuric acid medium and intercalates into the graphite structure (Figure S1), yielding defective black graphene layers [25]. The defective sites are also attacked by acids, which intercalate them. Gradually, amorphization proceeds, the interlayer distance between the graphene layers increases, the lattice parameter along the *c*-axis (axis perpendicular to the carbon layers) decreases, the number of layers reduces [26] and yields soluble and gaseous products.

**Figure 1:**
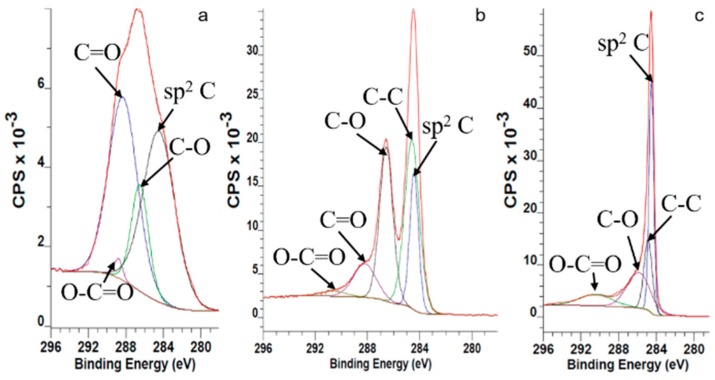
C1s XPS spectra of graphene oxide samples: (**a**) GO, (**b**) bwGO, (**c**) rGO and rbwGO.

When KMnO_4_ is added to the stage-1 GIC, which has a high intercalation potential in sulfuric acid, it starts to oxidize graphene layers thoroughly and gas evolution is observed when the formed graphite foam heats up to 35 °C. The synthesized graphite-intercalated suspension is not stable when it is in contact with water. These processes finish with the formation of different functional groups (phenolic –OH, lactone, quinone, ketone and carboxyl) at both peripheral edges and defects on the basal planes. Due to incomplete removal of sulfuric and nitric residuals trapped between the graphene layers [27], XPS and EDX results (Figure S2) showed characteristic bands for both sulfur and nitrogen. Sulfuric moieties with 1.06 atomic percentage (at.%) and nitric moieties with 0.14 at.% recorded by XPS confirm this hypothesis. Considering the complete oxidation of stage-1 GIC into graphite oxide, the amount of the recorded sulfuric and nitric moieties are negligible. The C1s XPS spectrum of the GO samples is shown in Figure 1 As it is seen from this figure, all graphene samples exhibit four peaks. The peaks at around ~284.4 eV are assigned to C–C and C=C of graphene lattices. Epoxy and phenolic –OH attached carbons show a relevant binding energy at ~286.5 eV, while the corresponding signals of ketone, lactone and carboxyl groups containing carbons are located at ~288–290 eV. The signals of carbons from ketone groups located at the defect areas of the GO samples are located at ~285.6 and ~285.8 eV [28].

**Figure 2:**
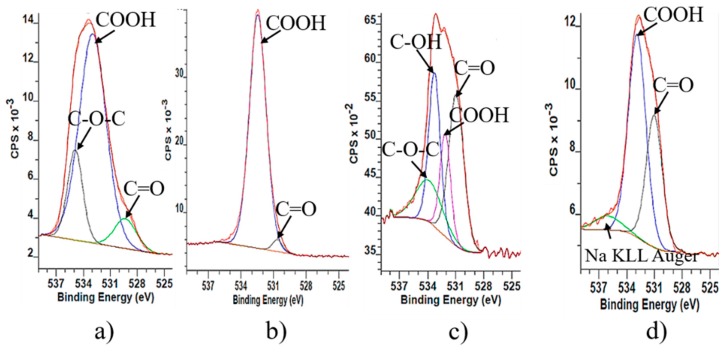
O1s XPS spectra of graphene oxide samples: (**a**) GO; (**b**) bwGO; (**c**) rGO and (**d**) rbwGO.

The results from O1s spectra of GO samples confirm the C1s results according to which the graphite structure is strongly oxidized and contains different oxygen functionalities. Therefore, the signal around ~530 eV corresponds to quinone, ~532 eV (C=O and O–C=O from carboxylic group), the signal around ~533 eV corresponds to phenolic –OH and epoxy groups, and ~534 eV to lactone groups (Figure 2). O1s XPS shows qualitatively that during reduction some amount of oxygen functionalities remain on the surface. They are mainly phenolic hydroxyl groups and epoxy groups. The reason for their incomplete reduction could be explained with the existence of oxidative debris on the surface of graphene oxide. However, during reduction hydrazine hydrate molecules react with oxidative debris and they are not able to reach the oxygen surface functionalities of the big layers. Furthermore, the reduction of base-washed graphene oxide (rbwGO) shows different result than reduced graphene oxide (rGO). On the surface of the rbwGO there is no assignment of the existence of epoxy and phenolic –OH groups. The possible surface functionalities of the rbwGO are quinone, ketone, lactone and carboxylic groups. 

**Figure 3:**
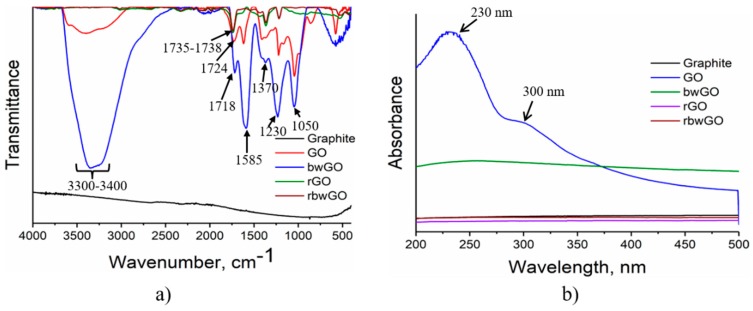
FTIR (**a**) and UV-Vis (**b**) spectra of GO, base-washed GO and their reduced forms.

Figure 3a shows the FTIR spectra of GO samples. It is clear from this figure that pristine GO (red color) has a transmission band near ~1620 cm^−1^, assigned to physisorbed water [29,30] by hydrogen bonds. The broad band at ~3300–3400 cm^−1^ denotes C–OH stretching vibrations. The high-wavenumber shoulder (~3600 cm^−1^) observed in the GO spectrum can be identified with the –OH stretching (high frequency) vibration of edge hydroxyl groups [30]. The C–OH stretching vibration in the base-washed graphene oxide (bwGO) spectrum is significantly higher than for GO and the physisorbed water peak disappeared. This indicates that in the basic medium (pH 14) all non-covalently adsorbed OD is stripped off from the surface of GO and carboxylic groups turned into the –COONa form. A reduction in the FTIR spectrum at ~3300 cm^−1^ indicates the high hydrophilicity of bwGO. Stretching vibrations of carbonyl groups observed between 1718 and 1738 cm^−1^ indicate the formation of carboxyl, quinone and six-membered lactone moieties after oxidation of graphite. When GO is freed from OD, a strong peak at 1585 cm^−1^ appeared showing conjugated benzene rings. –OH bending and C–O stretching vibrations from –COOH groups of the samples can be observed at 1410 or 1370 cm^−1^ and 1220 or 1230 cm^−1^, respectively. Epoxy group (C–O–C) stretching vibrations are found at 1045 and 1050 cm^−1^. After reduction with hydrazine hydrate, these peaks disappear.

The FTIR analysis of OD showed that it also contains oxygen functionalities. The broad band between 3300 and 3500 cm^−1^ attributes to C–OH stretching vibrations mainly located at the edges. The peaks at 1524, 1525, 1526 and 1558 cm^−1^ belong to benzene rings. The carbonyl (C=O) groups show the stretching vibrations at 1597, 1643 and 1673 cm^−1^, however the peak of lactone is at 1735 cm^−1^. –OH bending and C–O stretching vibrations of –COOH groups of OD can be observed at 1400–1425 cm^−1^ and 1230 cm^−1^, respectively. The FTIR spectra of OD is shown in Figure S3.

From UV-Vis spectroscopic studies (Figure 3b), it is seen that the dominant optical absorption of GO is located at ~230 nm. It belongs to the π-π* plasmon peak of nanometer-scale sp^2^ clusters and C=C chromophore units. The shoulder peak at ~300 nm corresponds to an n-π* plasmon peak of carboxyl, other carbonyl and hydroxyl auxochromes. The rest of the samples do not show any peaks, which is explained by reduction and substitution of oxygen functionalities. 

**Figure 4:**
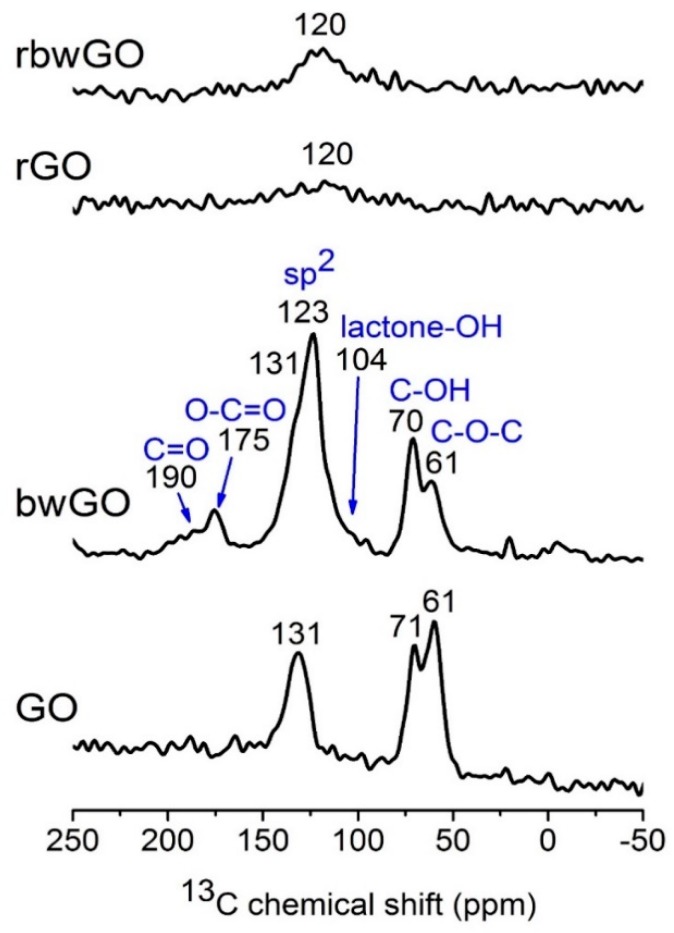
Direct excitation ^13^C MAS NMR spectra of graphene oxide samples.

Solid-state NMR experiments were performed to study the molecular structure of the GO samples (Figure 4). Direct excitation ^13^C magic angle spinning (MAS) NMR spectra of GO and bwGO exhibit similar features, showing three dominant signals at 60, 70 and ~130 ppm, which can be assigned to the epoxide (C–O–C), hydroxyl group (C–OH, most likely tertiary alcohol), and sp^2^ carbon of graphene, respectively. These characteristics are in agreement with previous reports, demonstrating that graphite is highly oxidized. In bwGO, additional weak signals occur at 104, 175 and 190 ppm. The signals at ~100 ppm can be attributed to a six-/five-membered lactone ring containing hydroxyl groups (lactol) along the periphery of the GO layer [31]. The signals at ~170 and 190 ppm are ascribed to carboxylic moieties that are bound to aromatic rings and ketone/quinone type parts of GO, respectively. Broad overlapping shoulder at ~110 and ~140 ppm are also present, which can be attributed to phenolic –OH group. Interestingly, two signals of sp^2^ carbon are observed at 120 and 130 ppm for bwGO, suggesting the presence of sp^2^ species in different local environments. ^13^C MAS NMR results of “washed” graphene oxides confirm that both H– and Na–form contain ketone, quinone, carboxyl, lactone with hydroxyl group (lactol), epoxy and C−OH functional groups, which are distributed around the sp^2^ graphene structure (Figure S4). To obtain further structural information, ^13^C{^1^H} CP MAS NMR spectra were measured (Figure S4). Since the CP efficiency depends on the dipolar interaction between nuclear spin pairs, which is inversely proportional to the cube of the internuclear distance, the CP signal can be selectively enhanced for carbon centers nearby protons. The relative intensity of the signal at 130 ppm with respect to that of 120 ppm is stronger in ^13^C{^1^H} CP MAS NMR spectrum than in the direct excitation spectrum, suggesting that the sp^2^ carbon of GO nearby protons (C–OH or trapped water between the layers) is responsible for the signal at 130 ppm. Moreover, the signals corresponding to lactone with -OH moieties are clearly visible in the CP spectra. In contrast, reduced GO showed very different MAS NMR spectra. For both reduced GO (reduced graphene oxide (rGO), and base-washed and reduced graphene oxide (rbwGO)), only a very broad sp^2^ signal is observed at 120 ppm, which is lower than that of GO and the signals corresponding to other oxidative moieties are not visible. Furthermore, tuning and matching of ^1^H and ^13^C channels to obtain MAS NMR spectra of reduced GO were extremely difficult and CP MAS NMR spectra could not be acquired. It is likely that the reduction procedure yields highly conductive materials, which are similar to pure graphite. However, it is not clear whether all the oxidative moieties are completely eliminated due to the poor spectral quality.

^13^C{^1^H} CP MAS NMR experiment for OD-1 shows that ketone, carboxylic and hydroxyl groups are the mainly distributed functionalities (Figure S5).

Elemental analysis shows that after oxidation, there is a moderate alteration in the weight percentage of the elements and the results are depicted in Table S1. Therefore, carbon being ~100% in graphite decreased to ~47 wt.% in GO, while oxygen increased by ~48 wt.%. The C/O ratio is 1.32 in the GO sample, which evidences the high level of oxidation of graphite. After stripping off the oxidative debris and reduction of graphene oxide, carbon increased to ~55, ~86 and ~79 wt.% and by contrast, the amount of oxygen decreased to ~39, ~10 and ~15 wt.% in bwGO, rGO, and base-washed and reduced graphene oxide(rbwGO), respectively. 

The C/O ratio reaches its highest level in reduced graphene oxide by ~12, which results from the loss of most oxygen containing functional groups.

Research on carbon blacks and other carbons conducted by Boehm considered that surface oxides are mainly at the periphery or at vacant defect sites of graphitic basal planes [32]. These surface functionalities can be divided into acidic and basic oxides: acidic oxides are bound to the edges of graphene layers, while basic groups can be distributed on basal planes. The plausible acidic groups on graphene layers can be carboxylic, lactonic and phenolic –OH groups [33], which can be quantitatively analyzed. Using a “standardized Boehm titration” method [22,23] the surface oxygen containing functionalities of the synthesized graphene flakes were calculated and the concentration of the carbon surface functional groups are given in Table 1.

As it is seen from Table 1, phenolic –OH groups decrease after washing and reduction, while lactone groups increase from GO to reduced forms. This data affirms the spectroscopic results where after reduction, hydroxyl groups disappear and lactone groups become more intense. The decrease of carboxylic groups in the sample of bwGO explains that at strong oxidizing conditions graphene layers break and form small graphene-like sheets that play a surfactant role when GO dispersions are prepared. In addition, the reduction of bwGO shows an almost two times increase in the amount of this group. It may be explained by the fact that during reduction some hydrazine moieties covalently linked to the graphene layers, which increases the absorption of reaction bases for acidic surface determination. Determination of basic surface functional groups with titration methods is complicated due to the existence of π electron clouds of benzene rings [34,35,36].

Raman spectroscopy is widely used to measure some of the properties of graphene samples. As a non-destructive spectroscopic method, Raman spectroscopy is a promising tool for the characterization of the 2D allotrope of carbon, in particular the characterization of "vacancy defects” [37]. Vacancy summarizes the absence of one or several carbon atoms from the hexagonal honeycomb graphene lattice, as well as, the edges of the structure (Figure S6). Pristine crystalline graphite shows only one Lorentzian G-band at ~1580 cm^−1^. When graphite has structural disorder, D, 2D, D + D’ and 2D’ bands are also activated [38]. In our measurements, graphite showed a G-band at ~1580 cm^−1^. It gives information about the E_2g_ phonon at the Brillouin zone center. A small D-band at ~1360 cm^−1^ attributes to the breathing mode vibration of six-atom benzene rings or it is activated by the boundary of the larger crystallinity, explaining the scattering of electrons from armchair edges [39,40]. 

**Figure 5:**
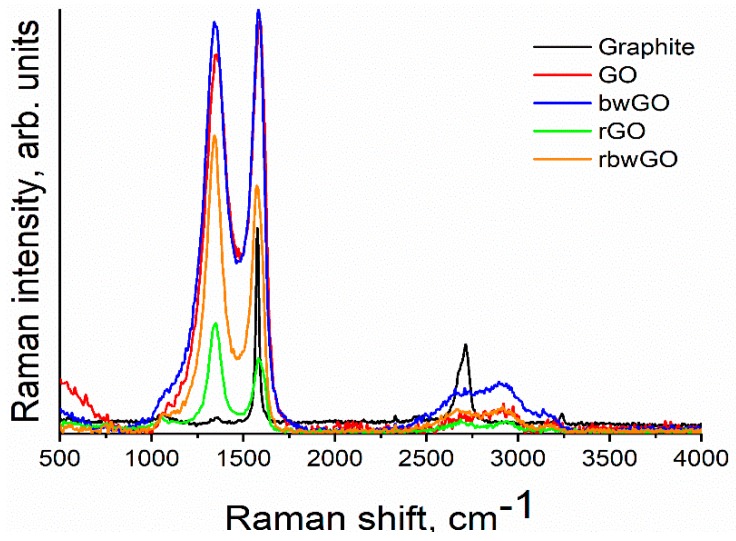
Raman spectra of graphene oxide samples.

After oxidation of graphite to conventional graphite oxide (CGO), the degree of order of the structure alters and the intensity of D-band increases in comparison with graphite, and the G-band in case of reduced forms decreases (Figure 5). The D peak in oxidized forms lies between 1342 and 1356 cm^−1^. This shows that GO samples are in a distorted form of the sp^2^ crystal structure and contain an enormous quantity of defects. In general, the activation of the D peak occurs within 3–4 nm size regions of the crystals that are close to defects or an edge [40]. Another band which confirms the defect formation is the appearance of D+D’ band (~2920–2940 cm^−1^), corresponding to the backscattering of a phonon at a hole point and it is much broader than other bands. This peak is a significant evidence that distinguishes GO samples from graphite. No defects are required for the activation of 2D and 2D’ bands [41]. Therefore, the 2D peak (~2700 cm^−1^) shape can be useful for determination of the number and orientation of graphene layers. A single sharp peak at 2D-band peak is considered to be an existence of single-layer graphene. During experiments bulk form of graphene oxide samples were used and the results show that GO samples contain many single layers (Table S2). This result suggests that the synthesized graphene oxide samples need to be exfoliated in order to get single-layer graphene oxide layers.

**Figure 6:**
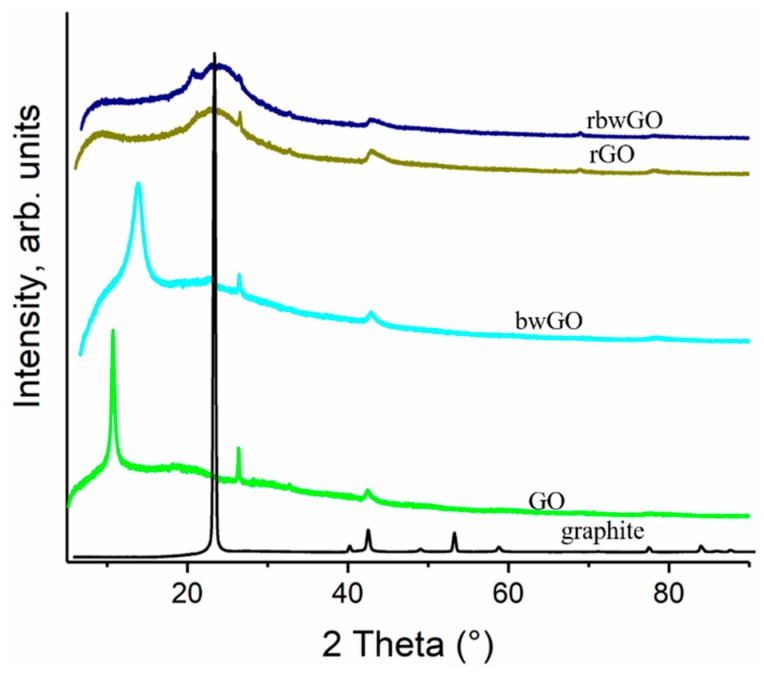
XRD patterns of graphene oxide samples.

Figure 6 depicts the XRD pattern of pristine graphite and GO samples. Graphite flakes exhibit a sharp 002 reflection at ~26.6°, corresponding to its interlayer spacing of 0.34 nm. Using the Scherrer equation for this sharp peak it is revealed that the thickness of graphite flakes is about 12.9 nm and the number of the layers in this crystal is 38. In case of GO and bwGO, the strong 2θ peak is around ~10.7° and ~13.6° showing a 001 reflection, which amounts to 0.83 and 0.65 nm in basal spacing, respectively. These explain the existence of oxygen functionalities increasing the interlayer distance between the layers. Furthermore, the XRD patterns of the reduced graphene oxide samples are different from GO, showing that the crystal sizes decreased by amorphization. This information confirms that the crystalline structure after oxidation is distorted, and these data correlate well with the Raman data (Table S3).

**Figure 7:**
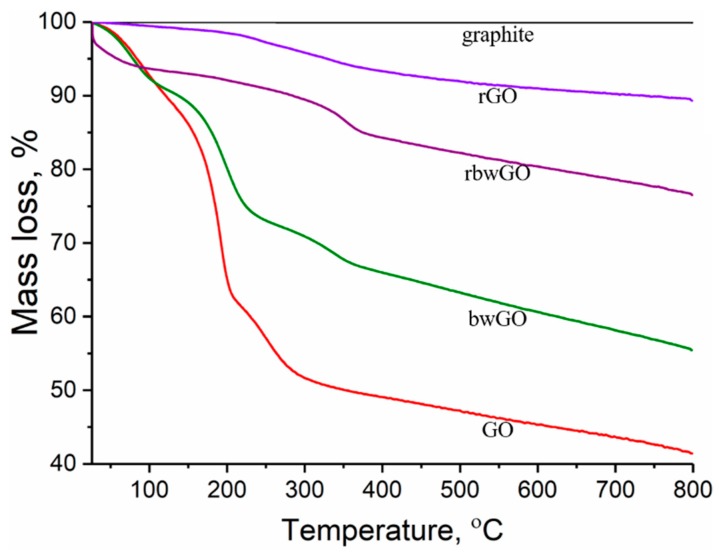
Thermogravimetric results of graphene oxide samples.

Testing the thermal stability is also one of the methods to investigate the existence of oxygen containing functionalities through the degradation of the investigated samples. In order to evaluate the oxygen containing functional groups of GO samples thermogravimetric analysis was performed under argon atmosphere. Figure 7 shows the mass loss of the samples as a function of temperature. It is clear that graphite does not show any mass loss, thus explaining why there are no oxygen containing functionalities. After oxidation, the graphite structure changes dramatically to CGO and contains an enormous amount of oxygen containing functionalities. Experiments revealed that GO and bwGO samples degrade mainly in three steps, which show the high degree of oxidation. The first step (26–125 °C) of ~10% mass loss belongs to evaporation of adsorbed water which is located between the lamellar layers of CGO [42]. The major mass loss is between 130 and 220 °C by ~28% in GO and by ~18% in bwGO. In this range, decomposition occurs with the evolution of water molecules from neighbouring hydroxyl groups, and carbon dioxide and carbon monoxide from carboxylic and lactone groups [43]. In comparison with these results, reduced graphene oxide samples are stable at this stage. As for GO and bwGO, a sharp weight loss (between 30 °C and 100 °C) in rbwGO sample is explained by the evaporation of trapped water molecules between the graphene layers. The third degradation range (220–400 °C) by ~12% weight loss in CGO and ~6% mass loss in the other graphene oxide samples could be explained by decomposition of quinone and ketone groups. Thermogravimetric analysis supported the thesis of Eigler et al. [44] that the small amount of sulfur recorded by XPS attributes to a covalently linked organosulfate moiety rather than a trapped sulfur species between the graphene layers, which besides quinone and ketone groups also decompose. After base-washing, those moieties reduce from the surface of the GO which lead to the wrong interpretation of “partial reduced GO” [44]. In other GO samples, we clearly see the decomposition of oxygen functionalities.

**Figure 8:**
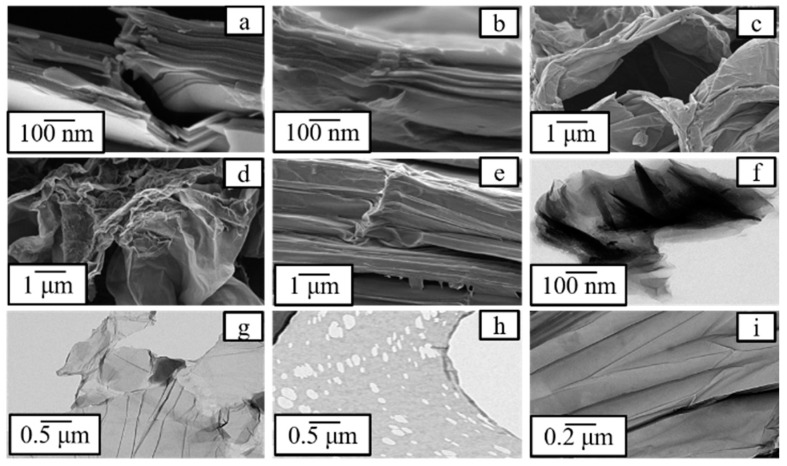
SEM (**a**–**e**) and TEM (**f**–**i**) images of graphene oxide and oxidative debris samples: (**a**)—graphite, (**b**)—GO, (**c**)—bwGO, (**d**)—rGO, (**e**)—rbwGO, (**f)**—bulk GO, (**g**)—single layer GO, (**h**)—oxidative debris, (**i**)—stacked and wrinkled GO layers.

Figure 8 shows scanning and transmission electron microscopic (SEM, TEM) images of GO and OD samples, respectively. The images prove that during graphite oxidation the number of layers reduces, crystallinity decreases and amorphization occurs. TEM images show that the obtained GO is a single-layer product and contains “more defective” OD (Figure 8h).

Exfoliation experiments were conducted for the analysis of the effect of oxidative debris [45] on agglomeration of graphene layers. After preparation of 10 dispersions with different concentrations, the correlation curves for the GO and bwGO samples were plotted (Figure S8). The plots were used for the calculation of the concentrations of graphene oxide dispersions. After centrifugation it was found that, after OD-stripping, the graphene oxide layers are more stable than the OD-containing graphene oxide. An explanation could be that OD non-covalently adsorbed on graphene oxide layers and attracts more solvent molecules for a stable dispersion. Therefore, the existence of oxidative debris on graphene oxide layers leads to hydrogen bonding between the layers, and agglomeration starts. Therefore, during experiments it was revealed that at 0.01 wt.% concentration the samples showed different concentrations after centrifugation. Hence, oxidative debris interacted between themselves, and the graphene layers became unstable. The concentration of OD-containing graphene oxide sample was 0.005 wt.%, while the concentration for OD-stripped graphene oxide layers was higher, with a concentration of 0.007 wt.%. The result of this experiment suggests that graphene oxide needs further purification after synthesis.

**Figure 9:**
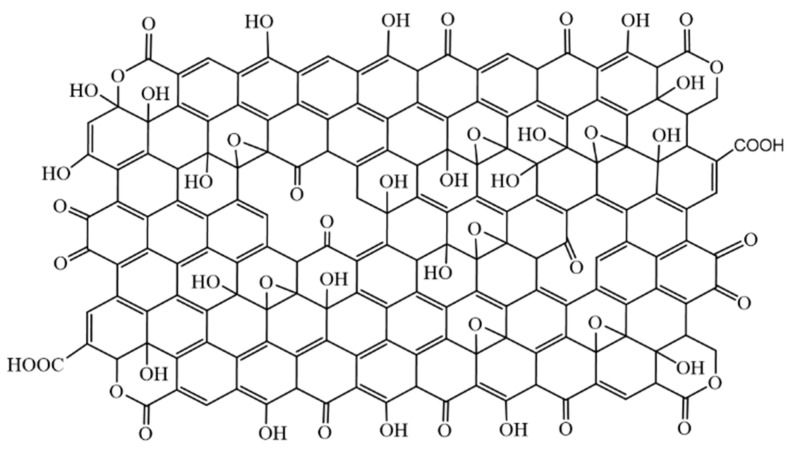
Proposed structure of graphene oxide layer.

In Figure 9 and Figure S7 we propose a new structural model for GO nanolayers and OD with respect to the obtained results. The analysis shows that graphene oxide layers also contain ketone groups in some areas; ketone and hydroxyl groups are close enough to each other to show keto-enol tautomerism [46]. It is found that GO layers also contain lactone groups. 

## 4. Conclusions

Thorough analysis showed that graphene oxide layers contain a large variety of oxygen functionalities. During synthesis by oxidation reaction, the interlayer distance increases, crystallinity decreases, the number of layers reduces, and soluble oxidative debris and gaseous products are obtained. Titration methods revealed that GO layers contain acidic surface oxygen functionalities, but the basic functional group analysis was not performed due to the possibility of experimental errors which could arise from the π electron cloud of benzene rings. Spectroscopic tools showed that on graphene layers there are also lactone, quinone and ketone sites located mainly at peripheral and defect areas. The suggested structural model explains the existence of NMR peaks corresponding to lactones. The lactone groups have been confirmed also by the standardized Boehm titration method. Experiments showed that in some regions the ketone groups are involved in keto-enol tautomerism with aromatic hydroxyl groups. Based on the obtained results, new possible structures for GO layers and for OD are implemented, the latter acting as a natural surfactant which stabilizes GO layers in dispersions. Unlike previous structural models, our model gives answers to the remaining questions from the Lerf–Klinowski and Lee models mentioned in the introduction. Experiments showed that the small amount of sulfur moiety covalently linked onto the graphene oxide surface is organosulfate, which is negligible for the proposed GO model. The proposed model does not abate the importance of the Lerf–Klinowski model; however, our model makes corrections on the weak points of the formerly proposed structure models. The newly proposed structure may be interesting for those researchers who work on graphene synthesis and functionalization and, moreover, for material scientists in order to prepare new types of composite materials.

## Figures and Tables

**Table 1:** Carbon surface functionalities (*n*_CSF_) of graphene oxide samples.

Samples	*n*_CSF_ ± SD (µmol/g)		
	Phenolic	Lactonic	Carboxylic
Reaction bases	0	0	0
GO	1017	565	5626
rGO	0	2747	0
bwGO	985	2009	216
rbwGO	0	2896	465

## Supplementary Materials

The following data is available online at https://www.mdpi.com/2079-4991/9/8/1180/s1, Figure S1: Intercalation, oxidation and exfoliation of graphene oxide layers, Figure S2: EDX results for graphene oxide samples, Figure S3: FTIR spectra of oxidative debris, Figure S4: Direct excitation ^13^C MAS NMR and ^13^C{^1^H} CP MAS NMR spectra, Figure S5: CP MAS spectrum of oxidative debris, Figure S6: Lattice models for graphene and graphene oxide, Figure S7: Possible structure of oxidative debris, Figure S8: Correlation curves for GO and bwGO exfoliation studies, Table S1: Elemental composition of graphene oxide samples, Table S2: Raman results for graphene oxide samples, Table S3: XRD analysis results.

## References

[1] Li D., Muller M.B., Gilje S., Kaner R.B., Wallace G.G. (2008). Processable aqueous dispersions of graphene nanosheets. Nat. Nano.

[2] Ahmad H., Fan M., Hui D. (2018). Graphene oxide incorporated functional materials: A review. Compos. Part B Eng..

[3] Perrozzi F., Prezioso S., Ottaviano L. (2014). Graphene oxide: From fundamentals to applications. J. Phys. Condens. Matter.

[4] Bouša D., Friess K., Pilnáček K., Vopička O., Lanč M., Fónod K., Pumera M., Sedmidubský D., Luxa J., Sofer Z. (2017). Thin, high-flux, self-standing, graphene oxide membranes for efficient hydrogen separation from gas mixtures. Chem. A Eur. J..

[5] Chi C., Wang X., Peng Y., Qian Y., Hu Z., Dong J., Zhao D. (2016). Facile preparation of graphene oxide membranes for gas separation. Chem. Mater..

[6] Li H., Song Z., Zhang X., Huang Y., Li S., Mao Y., Ploehn H.J., Bao Y., Yu M. (2013). Ultrathin, molecular-sieving graphene oxide membranes for selective hydrogen separation. Science.

[7] Hofmann U., Frenzel A., Csalán E. (1934). Die konstitution der graphitsäure und ihre reaktionen. Justus Liebigs Ann. Chem..

[8] Ruess G. (1947). Über das graphitoxyhydroxyd (graphitoxyd). Mon. Für Chem. Und Verwandte Teile And. Wiss..

[9] Scholz W., Boehm H.P. (1969). Untersuchungen am graphitoxid. Vi. Betrachtungen zur struktur des graphitoxids. Z. Für Anorg. Und Allg. Chem..

[10] Lerf A., He H., Forster M., Klinowski J. (1998). Structure of graphite oxide revisited. J. Phys. Chem. B.

[11] Lee D.W., De Los Santos V.L., Seo J.W., Felix L.L., Bustamante D.A., Cole J.M., Barnes C.H.W. (2010). The structure of graphite oxide: Investigation of its surface chemical groups. J. Phys. Chem. B.

[12] Paredes J.I., Villar-Rodil S., Martínez-Alonso A., Tascón J.M.D. (2008). Graphene oxide dispersions in organic solvents. Langmuir.

[13] Dreyer D.R., Park S., Bielawski C.W., Ruoff R.S. (2010). The chemistry of graphene oxide. Chem. Soc. Rev..

[14] Feicht P., Eigler S. (2018). Defects in graphene oxide as structural motifs. ChemNanoMat.

[15] Verdejo R., Lamoriniere S., Cottam B., Bismarck A., Shaffer M. (2007). Removal of oxidation debris from multi-walled carbon nanotubes. Chem. Commun..

[16] Whitby R.L.D., Gun’ko V.M., Korobeinyk A., Busquets R., Cundy A.B., László K., Skubiszewska-Zięba J., Leboda R., Tombácz E., Toth I.Y. (2012). Driving forces of conformational changes in single-layer graphene oxide. ACS Nano.

[17] Rourke J.P., Pandey P.A., Moore J.J., Bates M., Kinloch I.A., Young R.J., Wilson N.R. (2011). The real graphene oxide revealed: Stripping the oxidative debris from the graphene-like sheets. Angew. Chem. Int. Ed..

[18] Hiradate S., Yonezawa T., Takesako H. (2007). Fine fractionation and purification of the fulvic acid fraction using adsorption and precipitation procedures. Soil Sci. Plant Nutr..

[19] Hummers W.S., Offeman R.E. (1958). Preparation of graphitic oxide. J. Am. Chem. Soc..

[20] Wang Z., Shirley M.D., Meikle S.T., Whitby R.L.D., Mikhalovsky S.V. (2009). The surface acidity of acid oxidised multi-walled carbon nanotubes and the influence of in-situ generated fulvic acids on their stability in aqueous dispersions. Carbon.

[21] Stankovich S., Dikin D.A., Piner R.D., Kohlhaas K.A., Kleinhammes A., Jia Y., Wu Y., Nguyen S.T., Ruoff R.S. (2007). Synthesis of graphene-based nanosheets via chemical reduction of exfoliated graphite oxide. Carbon.

[22] Goertzen S.L., Thériault K.D., Oickle A.M., Tarasuk A.C., Andreas H.A. (2010). Standardization of the boehm titration. Part i. Co2 expulsion and endpoint determination. Carbon.

[23] Oickle A.M., Goertzen S.L., Hopper K.R., Abdalla Y.O., Andreas H.A. (2010). Standardization of the boehm titration: Part ii. Method of agitation, effect of filtering and dilute titrant. Carbon.

[24] Sorokina N.E., Khaskov M.A., Avdeev V.V., Nikol’skaya I.V. (2005). Reaction of graphite with sulfuric acid in the presence of kmno4. Russ. J. Gen. Chem..

[25] Sorokina N.E., Maksimova N.V., Avdeev V.V. (2001). Anodic oxidation of graphite in 10 to 98% hno3. Inorg. Mater..

[26] Roy Chowdhury D., Singh C., Paul A. (2014). Role of graphite precursor and sodium nitrate in graphite oxide synthesis. RSC Adv..

[27] Dimiev A.M., Bachilo S.M., Saito R., Tour J.M. (2012). Reversible formation of ammonium persulfate/sulfuric acid graphite intercalation compounds and their peculiar raman spectra. ACS Nano.

[28] Fujimoto A., Yamada Y., Koinuma M., Sato S. (2016). Origins of sp3c peaks in c1s x-ray photoelectron spectra of carbon materials. Anal. Chem..

[29] Szabó T., Berkesi O., Forgó P., Josepovits K., Sanakis Y., Petridis D., Dékány I. (2006). Evolution of surface functional groups in a series of progressively oxidized graphite oxides. Chem. Mater..

[30] Zhang C., Dabbs D.M., Liu L.-M., Aksay I.A., Car R., Selloni A. (2015). Combined effects of functional groups, lattice defects, and edges in the infrared spectra of graphene oxide. J. Phys. Chem. C.

[31] Gao W., Alemany L.B., Ci L., Ajayan P.M. (2009). New insights into the structure and reduction of graphite oxide. Nat. Chem.

[32] Boehm H.P. (1994). Some aspects of the surface chemistry of carbon blacks and other carbons. Carbon.

[33] Boehm H.P. (2002). Surface oxides on carbon and their analysis: A critical assessment. Carbon.

[34] Montes-Morán M.A., Menéndez J.A., Fuente E., Suárez D. (1998). Contribution of the basal planes to carbon basicity:  An ab initio study of the h3o+−π interaction in cluster models. J. Phys. Chem. B.

[35] Suárez D., Menéndez J.A., Fuente E., Montes-Morán M.A. (1999). Contribution of pyrone-type structures to carbon basicity:  An ab initio study. Langmuir.

[36] Suárez D., Menéndez J.A., Fuente E., Montes-Morán M.A. (2000). Pyrone-like structures as novel oxygen-based organic superbases. Angew. Chem. Int. Ed..

[37] Zenkel C., Albuerne J., Emmler T., Boschetti-de-Fierro A., Helbig J., Abetz V. (2012). New strategies for the chemical characterization of multi-walled carbon nanotubes and their derivatives. Microchim. Acta.

[38] Kudin K.N., Ozbas B., Schniepp H.C., Prud’homme R.K., Aksay I.A., Car R. (2008). Raman spectra of graphite oxide and functionalized graphene sheets. Nano Lett..

[39] Tuinstra F., Koenig J.L. (1970). Raman spectrum of graphite. J. Chem. Phys..

[40] Ferrari A.C., Basko D.M. (2013). Raman spectroscopy as a versatile tool for studying the properties of graphene. Nat. Nano.

[41] Cançado L.G., Jorio A., Martins Ferreira E.H., Stavale F., Achete C.A., Capaz R.B., Moutinho M.V.O., Lombardo A., Kulmala T.S., Ferrari A.C. (2011). Quantifying defects in graphene via raman spectroscopy at different excitation energies. Nano Lett..

[42] Cai W., Piner R.D., Stadermann F.J., Park S., Shaibat M.A., Ishii Y., Yang D., Velamakanni A., An S.J., Stoller M. (2008). Synthesis and solid-state nmr structural characterization of ^13^c-labeled graphite oxide. Science.

[43] Alosmanov R., Wolski K., Matuschek G., Magerramov A., Azizov A., Zimmermann R., Aliyev E., Zapotoczny S. (2017). Effect of functional groups on the thermal degradation of phosphorus-and phosphorus/nitrogen-containing functional polymers. J. Therm. Anal. Calorim..

[44] Eigler S., Dotzer C., Hof F., Bauer W., Hirsch A. (2013). Sulfur species in graphene oxide. Chem. A Eur. J..

[45] Naumov A., Grote F., Overgaard M., Roth A., Halbig C.E., Nørgaard K., Guldi D.M., Eigler S. (2016). Graphene oxide: A one-versus two-component material. J. Am. Chem. Soc..

[46] Dimiev A.M., Eigler S. (2016). Graphene Oxide: Fundamentals and Applications.

