# Supplementary material for: Structural Characterization of Graphene Oxide: Surface Functional Groups and Fractionated Oxidative Debris
Source: Nanomaterials (Basel). 2019 Aug 18;9(8):1180. doi: 10.3390/nano9081180 (PMC6724119; doi:10.3390/nano9081180)
Supplement: Supplementary file 1 [file nanomaterials-09-01180-s001.pdf]

Supplementary Material:

Structural Characterization of Graphene Oxide: Surface Functional Groups and Fractionated Oxidative Debris

Elvin Aliyev [1], Volkan Filiz [1,*], Muntazim M. Khan [1], Young Joo Lee [2], Clarissa Abetz [1] and Volker Abetz [1,3]

[1] Helmholtz-Zentrum Geesthacht, Institute of Polymer Research, Max-Planck-Str. 1, 21502 Geesthacht, Germany
[2] Institute of Inorganic and Applied Chemistry, Department of Chemistry, University of Hamburg, Martin-Luther-King-Platz-6, 20146 Hamburg, Germany
[3] Institute of Physical Chemistry, Department of Chemistry, University of Hamburg, Martin-Luther-King-Platz-6, 20146 Hamburg, Germany

Contents

1. Synthesis and exfoliation of Graphene Oxide S2
2. EDX results for graphene oxide samples S3
3. FTIR spectra of oxidative debris S3
4. Direct excitation ^{13}C MAS NMR and $^{13}C\{^{1}H\}$ CP MAS NMR spectra S4
5. CP MAS spectrum of oxidative debris S4
6. Lattice models for graphene and graphene oxide S5
7. A table for elemental composition of graphene oxide samples S5
8. A table for Raman results for graphene oxide samples S5
9. A table for XRD analysis results S6
10. A proposed structure for oxidative debris S6
11. Correlation between absorbance and concentration of GO and bwGO dispersions S7

$$NaNO_3 + H_2SO_4 \longrightarrow NaHSO_4 + HNO_3$$

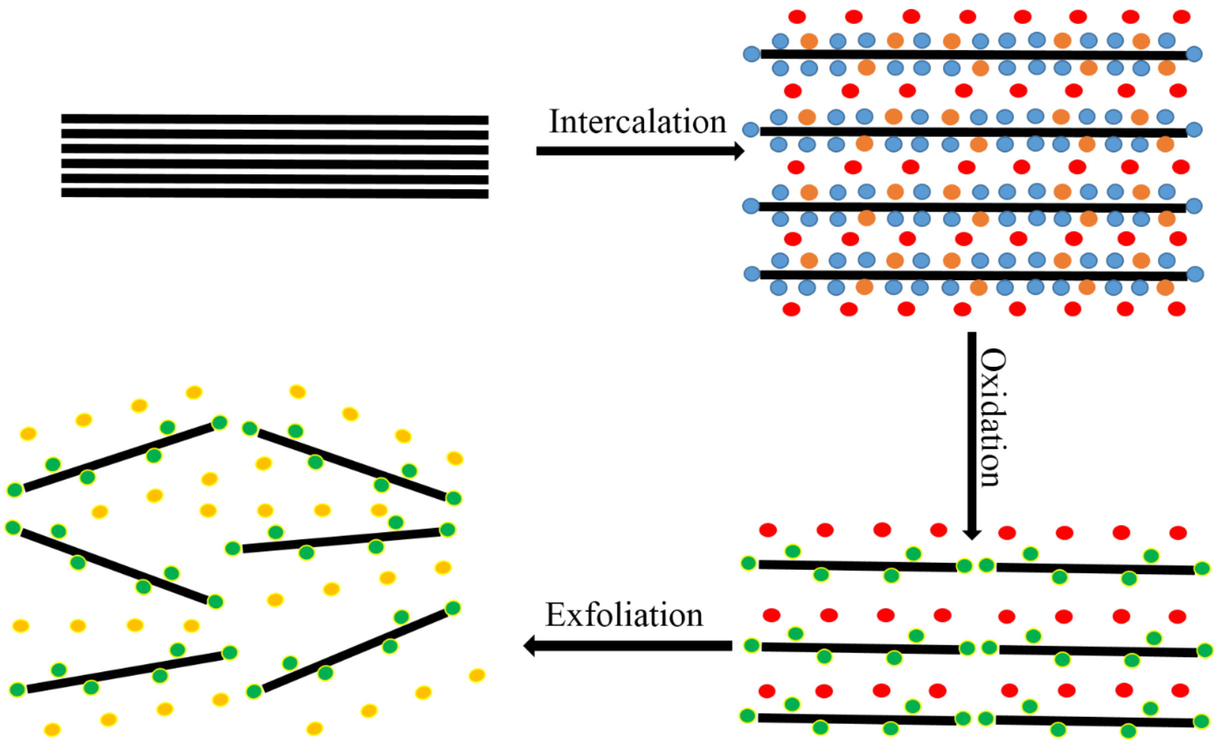

Figure S1. Intercalation, oxidation and exfoliation of graphene oxide layers: - $HSO4^-$, - NO_3^- ; - Na^+; - oxygen functionalities; - oxidative debris.

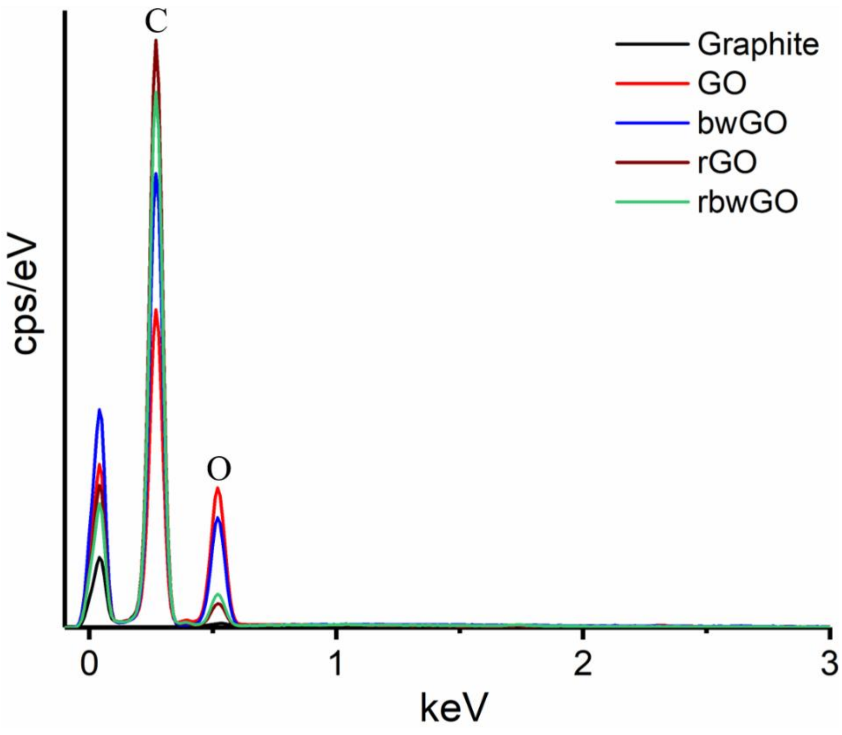

Figure S2. EDX results for graphene oxide samples.

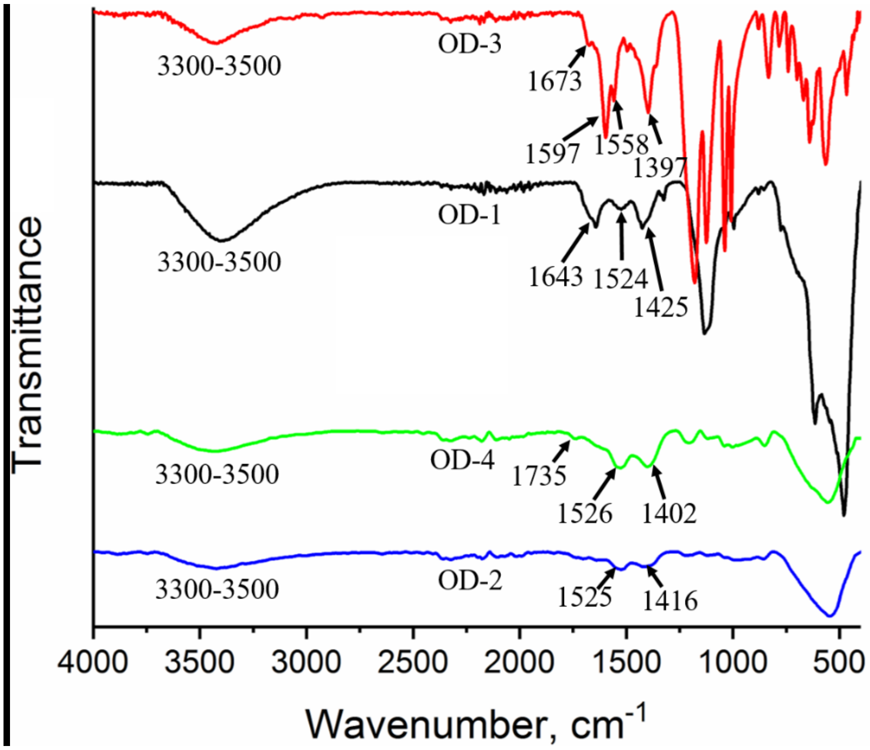

Figure S3. FTIR spectra of oxidative debris.

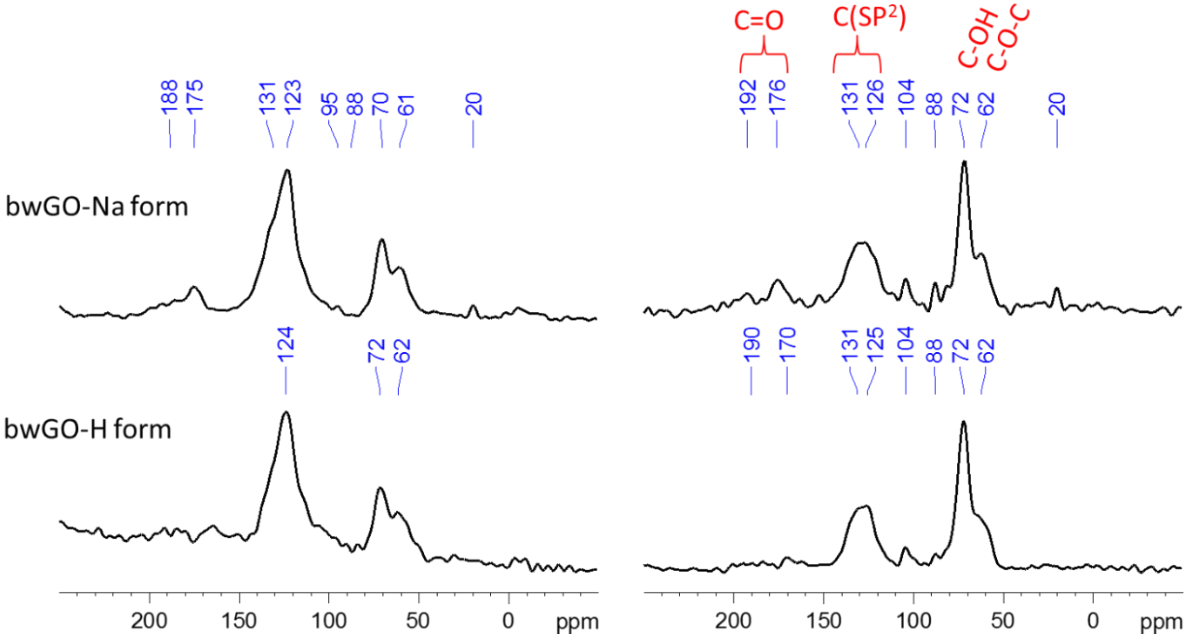

Figure S4. Direct excitation ^{13}C MAS NMR (left) and $^{13}C\{^1H\}$ CP MAS NMR (right) spectra of base-washed graphene oxide (sodium and hydrogen forms).

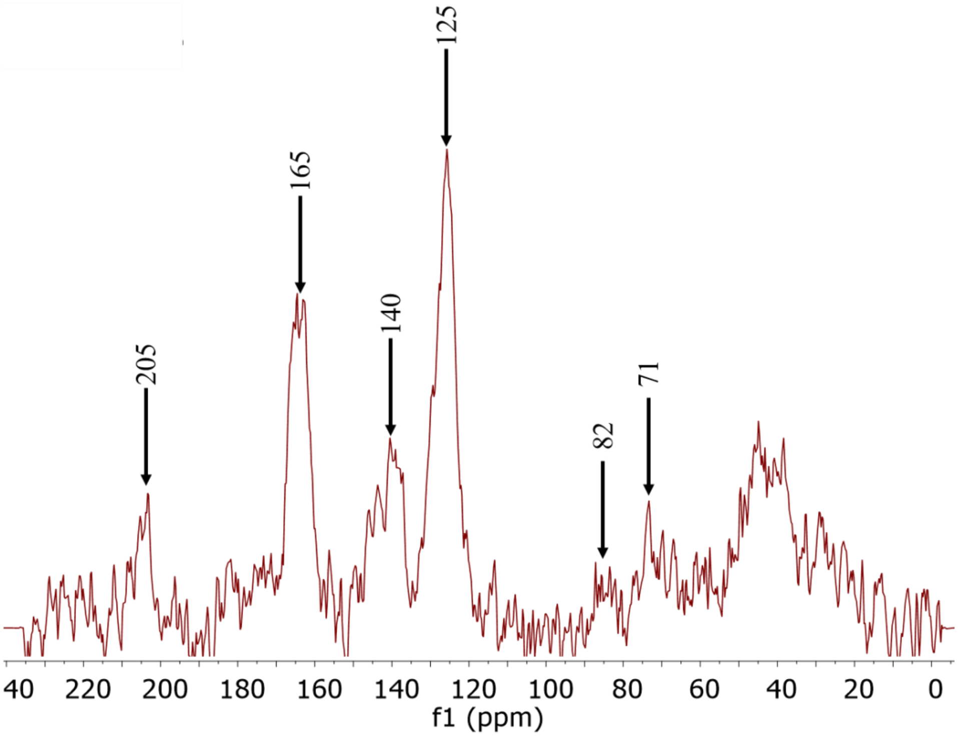

Figure S5. $^{13}C\{^{1}H\}$ CP MAS NMR spectrum of OD-1.

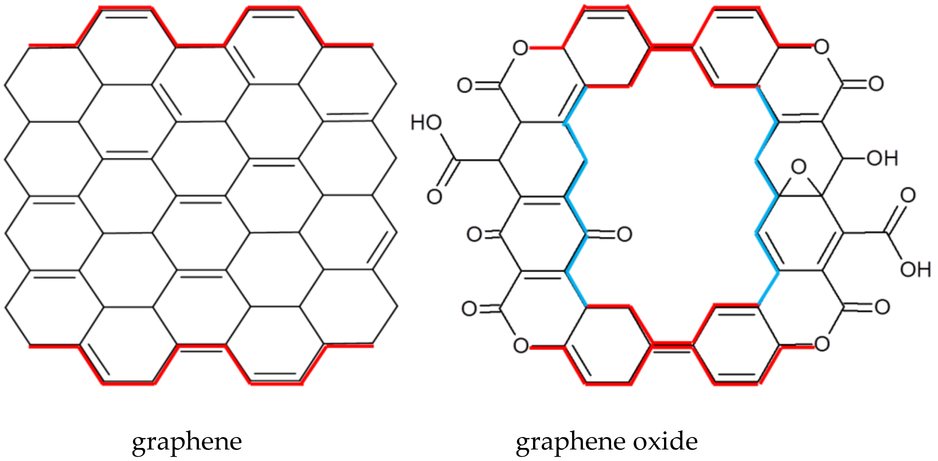

Figure S6. Lattice model for the edges and round hole. Red and blue lines mark armchair and zigzag edges, respectively.

Table S1. Elemental composition of graphene oxide samples.

Samples	Elements by % weight			C/O ratio	C/H ratio
	C	H	O		
Graphite	99.6	0.4	-	-	0.4
GO	47.1	4.4	47.6	1.32	0.89
rGO	85.5	0.84	9.6	11.9	8.48
bwGO	55.0	2.4	39.2	1.87	1.91
rbwGO	78.8	1.27	14.8	7.1	5.17

Table S2. Raman results for graphene oxide samples.

Samples	Raman peaks, cm^{-1}					ID/IG	I2D/IG
	D-band	G-band	2D-band	D+D'-band	2D'-band		
Graphite	1359	1578	2713	-	3240	~0.08	~5.6
GO	1356	1592	2683	2920	3204	~0.92	~0.05
rGO	1351	1584	2685	2939	-	~1.47	~0.19
bwGO	1342	1583	2708	2920	3183	~0.97	~0.11
rbwGO	1343	1575	2684	2916	3165	~1.2	~0.09

Table S3. XRD analysis results.

Samples	2θ max. (002)	FWHM(La)	La (nm)	d (nm)	N	2θ max. (100)	FWHM(Lc)	Lc (nm)
Graphite	26.6	0.66	12.9	0.34	38	44.6	0.66	13.6
GO	10.7	6.72	1.24	0.83	2	42.5	7.14	1.25
rGO	-	-	-	-	-	-	-	-
bwGO	13.8	11.37	0.74	0.65	1	42.9	13.19	0.68
rbwGO	-	-	-	-	-	-	-	-

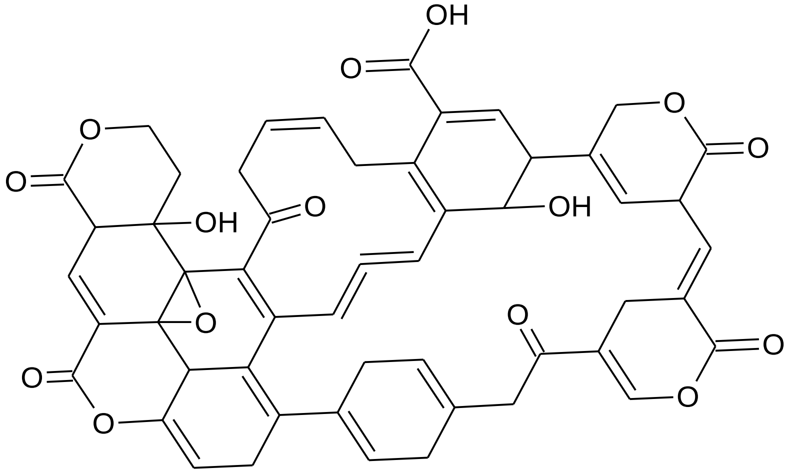

Figure S7. Possible structure of oxidative debris.

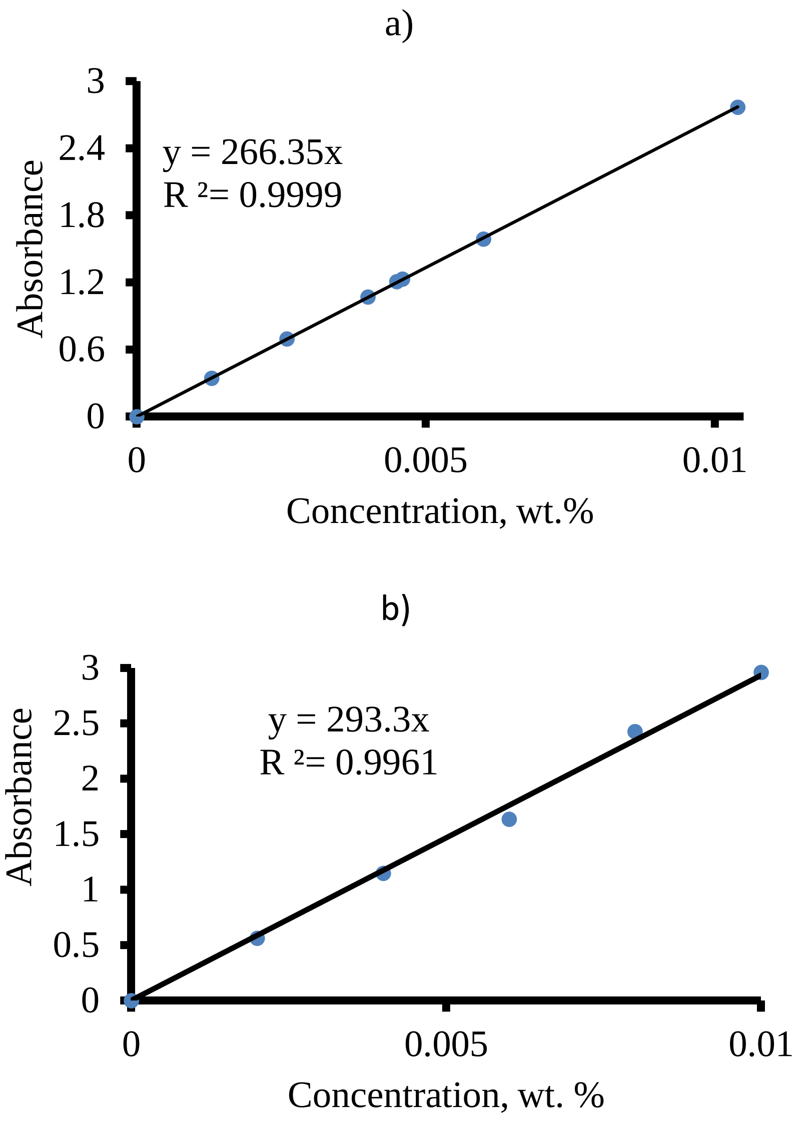

Figure S8. Correlation between absorbance and concentration of GO (a) and bwGO (b) solutions obtained at 600 nm on an UVmini – 1240 spectrophotometer.
